# Advancing 3D scaffold models for metastatic prostate cancer in bone: Materials, manufacture, and future perspectives

**DOI:** 10.1016/j.bbiosy.2025.100125

**Published:** 2025-11-20

**Authors:** Annachiara Dozzo, Aikaterini Dedeloudi, Dimitrios Lamprou, Caitriona M. O’Driscoll, Katie B. Ryan

**Affiliations:** a School of Pharmacy, University College Cork, T12 K8AF Cork, Ireland; b School of Pharmacy, Queen’s University Belfast, 97 Lisburn Road, BT9 7BL Belfast, UK; c SSPC Research Ireland Centre for Pharmaceuticals, School of Pharmacy, University College Cork, T12 K8AF Cork, Ireland

**Keywords:** Metastatic prostate cancer (mPC), Bone metastases, 3D cancer models, Scaffolds, 3D printing, Drug screening tools

## Abstract

The metastatic spread of prostate cancer to the bone is debilitating for patients and is associated with a poor prognostic outlook. Gaining a deeper insight into the pathophysiology of prostate cancer and its metastatic spread using advanced, in vitro, 3D models could enhance disease understanding and the successful development of new drug treatments. The in vitro replication of prostate cancer metastasisation to bone is challenging and has received little research attention. The 3D scaffold models investigated to date exhibit different physical characteristics and utilise a diverse range of materials, and manufacturing methods. This variability is likely driven by the lack of standardisation and guidance. Herein, we review the scaffold models used as in vitro bone niches of prostate cancer metastases and examine the different physical (e.g. porosity, pore geometry) and material criteria that guide the design and production of artificial 3D bone aliases. We critically review different manufacturing techniques ranging from standard techniques (e.g. freeze drying) to more advanced additive manufacturing strategies that enable the production of more intricate and controlled structures. Ultimately, we provide insights and future directions to guide researchers investigating this poorly studied field.

## Highlights

• Biological and environmental cues in the bone contribute to prostate cancer cell colonization of the bone.
• Generation of 3D models with different materials leads to differing physical, and mechanical properties, impacting their predictive power.
• Conventional techniques e.g., lyophilization give rise to variability in 3D model properties.
• Advanced manufacture including 3D-printing, offer the potential to design and manufacture more intricate scaffold structures with enhanced reproducibility.

## Graphical abstract

**Figure:**
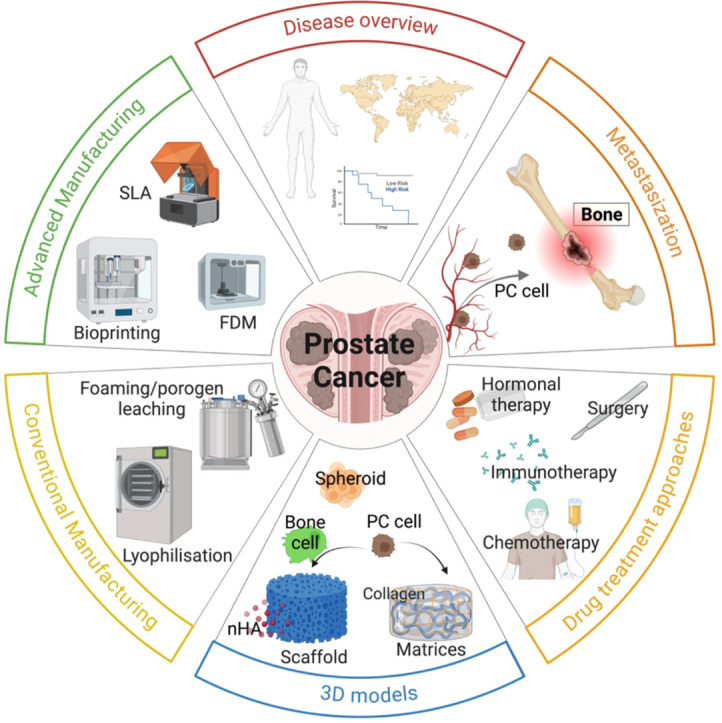

## 1 Introduction

Prostate cancer (PCa) is the most commonly diagnosed cancer in men and the second leading cause of cancer-related deaths in men in the United States [1]. In 2025, it is estimated that PCa will result in approximately 313,780 new cases, which represents nearly 30 % of all new cancer cases in men, and 35,770 deaths across the USA [1,2]. Each year, approximately 6.8 % of patients are expected to be diagnosed with metastatic spread to other sites [3,4]. PCa commonly metastasises to the bone, lymph nodes, liver, lungs, and the brain [5]. The bone, especially the axial skeleton and pelvis, represents preferential homing sites for PCa cells due to their proximity and rich reservoir of nutrients [6]. Bone metastases in metastatic prostate cancer (mPC) are associated with a poor prognosis with only 31 % of patients expected to survive beyond 5 years [3,7]. This is partly due to treatment resistance and the bone microenvironment’s capacity to modulate tumour behaviour and response to treatment [8,9].

To improve patient outcomes and develop more effective treatments, more physiologically relevant in vitro models are needed to study the behaviour of mPC cells and their interplay with the native bone environment. Conventional, 2D cell culture models fail to recapitulate the native cellular microenvironment or the disease process in vivo [10]. Consequently, this limits their predictive value in assessing the clinical potential of new drug candidates and contributes to the high rate of therapeutic failure [11].

The emergence of 3D in vitro models offers exciting opportunities to better replicate the native tissue environment, enhance understanding of diseases such as cancer, reduce the reliance on animal models in drug development and improve the screening of new drug treatments [12,13]. Despite the progress in modelling various tissue types in the last decade, a limited number of 3D scaffold models replicating bone structure and composition have been developed. Fewer still have been developed to model mPC spread to the bone. Indeed, accurately replicating bone tissue in vitro is challenging [14]. The lack of regulatory guidance and standardised design specifications for different tissue types has led to the development of diverse models employing a multitude of materials and manufacturing techniques. Consequently the resulting constructs have variable features and many fail to mimic the structural hierarchy and compositional characteristics of native bone tissue [15].

Recently, several reviews have been published focusing on the state-of-the-art in PCa 3D models [[16], [17], [18]], highlighting the advantages and drawbacks of the models and summarising the different materials and properties, (e.g., tunability, stiffness, processing techniques) employed. Prior reviews also provide an in-depth analysis of how the various 3D-models can affect cell behaviour, proliferation rate and gene expression [16,18]. However, the field lacks a critical review on the design and development of 3D models recapitulating bone and mPCa spread to the bone, in particular [19].

In this review, we examine the pertinent considerations for developing in vitro 3D scaffolds as models of bone and mPC including structural properties, material features and manufacturing techniques. We discuss the structural and compositional characteristics of bone alongside prostate cancer progression to identify the biological inspired cues that can guide 3D scaffold model development. Conventional techniques e.g., lyophilisation, and the associated advantages and limitations are discussed. We also explore advanced manufacture (AM) approaches including 3D-printing and computer-aided design (CAD), which offer the potential to design and manufacture more intricate scaffold structures with enhanced reproducibility. Finally, we examine the research to date investigating AM techniques to manufacture 3D scaffolds for modelling mPC and consider future directions in bioprinting.

## 2 Bone structural and cellular composition

**Fig. 1:**
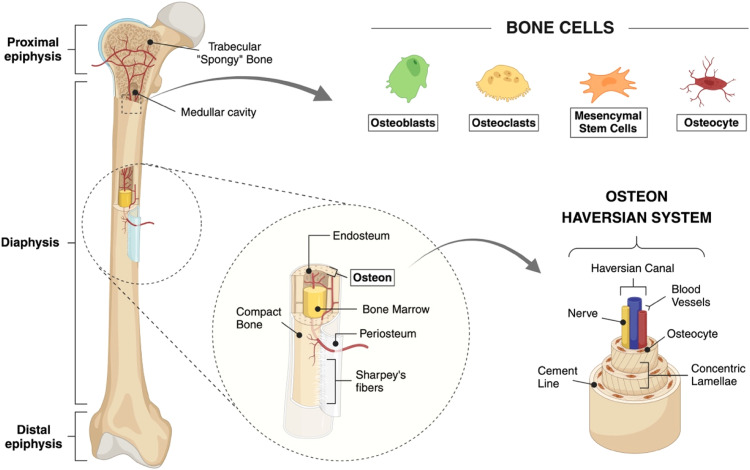
Schematic representation of a femur and its hierarchical structure. (**Top**) Key cell types include osteoblasts, osteocytes and osteoclasts and MSCs as progenitors play important roles in bone remodelling and homeostasis. These cells, when coordinated, remove old, damaged bone and replace it with new bone matrix. (**Left**) The structure of a long bone consists of proximal (top) and distal (bottom) epiphysis separated by a long diaphysis. (**Centre**) The cross section of the diaphysis shows the different layers composing the bone. Cancellous bone has a spongy, porous appearance, characterised by trabecular struts filled with bone marrow (BM) and interspersed with blood vessels. It is typically found in the epiphysis and metaphysis of long bones. The periosteum is the layer at the surface of the bone to which it is anchored via perforating Sharpey’s fibres. The endosteum lines the boundary between the marrow cavity and the compact bone. (**Right**) The compact bone consists of concentric tubular units, the Haversian systems or osteons running longitudinally in the bone. The hollow central canal in the osteon hosts nerve and blood vessels for nutrient supply. The surrounding lamellae are orientated to confer rigidity and high resistance to mechanical loads. Redrawn and modified from [28]. Created with BioRender.com.

The adult human skeleton comprises approximately 206 bones [20] that provide support, facilitate movement, protect vital organs, and maintain mineral homeostasis and haematopoiesis within the marrow space [21]. Bones anatomically consist of cortical (compact) and cancellous (trabecular) portions and both are similarly composed of organic extracellular matrix (ECM), mainly including collagen, and inorganic, carbonated hydroxyapatite (HA) minerals. However, they differ in their architecture and the density of matrix deposition. Cancellous bone is less dense, with a porous structure of trabecular struts that is filled with bone marrow (BM) and blood vessels. It is typically found in the epiphysis and metaphysis of long bones, or located in the middle regions in short, flat (e.g. pelvis, ribs) and irregular bones [[22], [23], [24]]. In contrast, cortical bone is denser and owes its rigidity to tightly packed cylindrical osteons (Fig. 1) [22,25]. The concentric lamellae are orientated to help confer rigidity and high resistance to mechanical loads. These differences in architecture, density and porosity between cortical and cancellous bone have important implications for 3D in vitro model design. The outer surface of the bones is covered by the periosteum, which comprises a superficial fibrous layer and a deeper cambium layer with mesenchymal stem cells (MSCs), osteoblasts, and fibroblasts [26]. While the cortical bone and the marrow cavity are separated by the endosteum, a thin layer of connective tissue [27].

Bone contains a dynamic population of cell types that are influenced by a variety of mechanical and biochemical signals including mechanical stimuli and changes in pH, influencing bone homeostasis [29,30]. While bone cells only account for a small proportion of bone volume, they are pivotal in bone formation and remodelling. Key cell types include osteoblasts, osteocytes and osteoclasts and MSCs as progenitors, which differ in their morphology and function [20,21,31]. MSCs may reside in bone marrow, vasculature, trabecular bone, periosteum, or bone canals, and can differentiate into osteoblasts or adipocytes according to environmental cues [32]. Osteoblasts, are cuboidal and synthesize organic bone matrix, regulate calcium and phosphate balance, and influence osteoclast activation under parathyroid hormone (PTH) control [33,34]. Osteoblasts can differentiate into bone-lining cells that interact with osteocytes, playing roles in bone remodelling and hematopoietic stem cell (HSC) differentiation [35,36]. Osteocytes, account for 90–95 % of bone cells, and vary in morphology depending on their location in the bone. They act as mechanosensors and modulate osteoclast recruitment contributing to bone remodelling [37]. Osteoclasts, are multinucleated cells that are derived from HSC through the myeloid pathway [38,39] and resorb bone through acidification and enzyme secretion [40]. Osteoclastogenesis, the process of osteoclast genesis and maturation, is regulated by RANKL binding to the RANK receptor on osteoclast precursor cells of the monocyte-macrophage lineage and represents a key regulator of bone resorption. RANKL is secreted by stromal cells, osteoblasts, and osteocytes. The soluble decoy receptor, osteoprotegerin (OPG), secreted by osteoblast lineage cells, can bind with RANKL, thereby inhibiting RANKL-RANK binding and osteoclast formation [41]. Dysregulation of the RANK/RANKL/OPG pathway can contribute to abnormal bone remodeling, which is characteristic of some bone diseases and skeletal metastases [42].

## 3 Metastatic spread of prostate cancer to bone

**Fig. 2:**
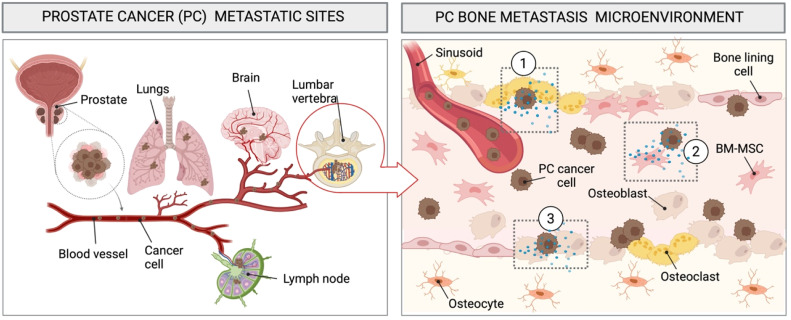
Schematic representation of prostate cancer (PCa) metastatic spread and bone colonisation. (Left) PCa cells spread to sites such as the lungs, brain, and bones via hematogenous or lymphatic routes. Particularly PCa cells can metastasize to bone sites e.g., the lumbar region of the spine. (Right) Within the bone, PCa cells elicit different bone responses and increase their metastatic potential depending on the interplay with resident cells in the bone microenvironment including amongst others, 1) osteoclasts, 2) bone marrow mesenchymal stem cells and 3) osteoblasts. Created with BioRender.com.

Prostate cancer can metastasize from the primary site in the prostate, and colonise other tissues or organs giving rise to metastases [43] at secondary sites including the bone, lung and the brain [44]. In the case of metastatic spread to the bone, the vertebrae in the lumbar spine and the pelvis, both part of the axial skeleton, are commonly affected due to their proximity (Fig. 2 **left panel**) coupled with bone’s rich vascularisation and variety of microenvironmental cues that attract and support cancer progression. PCa typically spreads via the hematogenous or lymphatic routes [45,46], with hematogenous spread contributing to metastases in the axial skeleton [6]. Bone metastases typically develop after years of disease progression and approximately 80 % of the patients with advanced PCa develop bone metastases, while approximately 10 % present with metastatic lesions early in the disease [[47], [48], [49]].

The ‘seed and soil’ theory proposed by Paget, fundamentally describes the metastatic process by which cancer cells (seeds) spread in search of the perfect microenvironment that provides the necessary factors for growth (soil) (Paget, 1989) [50]. The metastasisation process is indeed more complex and continues to be investigated [51]. The metastasisation of prostate cancer is a multi-step process which can be summarized by (i) cancer cell dissemination, (ii) localisation to the bone under the influence of environmental and chemotactic cues, (iii) attachment to the bone marrow endothelium, (iv) colonisation of the bone marrow compartment, (v) cancer cell adaptation (e.g., dormancy) to the bone microenvironment, (vi) re-activation of the proliferative state and (vii) remodelling of the bone environment [[52], [53], [54]].

Several mechanisms are reported to be involved in cancer cell dissemination. Wnt β-Catenin signalling is implicated in circulation, and migration, stemness and tumorigenesis potential [55]. Recent evidence suggests the reversible adaptive mechanisms of epithelial-to-mesenchymal and endothelial-to-mesenchymal transition may contribute to PCa dissemination [56,57] and hormone targeted treatment resistance [58]. The bone marrow provides a fertile environment for circulating cancer cells due to its vascular supply and abundance of growth factors. The exact niche colonised by PCa cells is debated, with evidence supporting both the endosteum, site of the osteoblast niche [59], and the HSC niche [60]. The lack of a physical boundary separating these niche environments suggests a complex interplay between PCa cells and the resident cells in the bone marrow microenvironment.

There are many drivers of PCa progression and cell homing to the bone. The stromal cell-derived factor 1(SDF-1)–CXCR-4 pathway is reputed to be involved in the processes of homing and invasion of PCa metastases to the bone [61]. PCa cells express the CXCR-4 receptor and migrate in response to the chemokine, SDF-1, which is expressed by osteoblasts and endothelial cells in the bone [61].

Bone marrow mesenchymal stem cells (BM-MSCs) are also implicated in PCa metastasis. Luo et. al. demonstrated that BM-MSCs secrete cytokines (e.g., CCL5) that downregulate the androgen receptor signalling pathway and enhance metastatic potential [60]. BM-MSCs may also contribute to microenvironmental adaption through secretion of a series of factors such as transforming growth factor beta 2 (TGF-β2) and thrombospondin 1 (TSP1) or exosomes rich in ‘quiescence inducing’ microRNAs, which regulate PCa cell dormancy, and has implications for PCa reactivation and relapse [62,63].

PCa cells can anchor and colonise extracellular matrix via integrin-mediated adhesions [64]. In the bone, PCa cells can interact with the local bone microenvironment, establish a mutual collaboration with the resident cells (osteoblasts and osteoclasts), disrupt normal bone remodelling and support tumour progression [65]. mPC is typically associated with osteoblastic (sclerotic) lesions, but mixed lesions and occasionally osteolytic lesions are seen. Osteoblastic lesions occur due to the preponderance of growth factors e.g., TGF-β, which promotes the deposition of new bone matrix [66,67]. PCa cells promote bone-formation through the secretion of bone morphogenetic proteins (BMPs) [67]. PCa cells secrete Endothelin-1 (ET-1), which actives osteoblasts via the endothelin A receptor (ET_A_R), further promoting bone formation [68]. Osteoblastic lesions are characterized by enhanced mineralization and abnormal matrix deposition [69].

Disruption of key signalling pathways including RANK–RANKL also contributes to altered bone remodelling [[70], [71], [72], [73]]. PCa cells express parathyroid hormone–related protein (PTHrP), which stimulates osteoblasts to secrete RANKL [52,74,75]. RANKL binding to RANK on osteoclasts triggers osteoclast-mediated bone resorption [76], which contributes to mixed lesion phenotypes. The process causes loosening of the bone tissue and release of biological factors such as TGF-β and insulin growth factor 1 (IGF-1) and increases extracellular calcium concentration [6], which is purported to attract more PCa cells to the site, and perpetuate a vicious cycle [6].

To gain deeper insights into the complex biology of mPC, there is a need for physiologically relevant and robust models that accurately replicate the multicellular interactions within the bone niche. However, very few examples exist in the literature. A notable, recent example includes a patient-specific, microphysiological system engineered using the LumeNEXT microfluidic platform to recapitulate the PCa metastatic bone microenvironment [9]. Seven different cell types including bone stromal and immune cells were embedded in a matrix of collagen together with prostate tumour epithelial spheroids. Additionally, a lumen lined with endothelial cells was integrated to simulate vascular structures. Model validation using standard-of-care drug treatments, darolutamide and docetaxel, revealed a reduced cytotoxic effect, emphasising the role of the bone tumour microenvironment in modulating therapeutic outcomes.

## 4 Treatment of prostate and metastatic prostate cancer

Different treatment approaches can be used to treat PCa, but the choice depends on the development stage of the disease and other factors such as the patient’s age, risk level and co-morbidity factors. When PCa is localized, initial management involves active surveillance to assess behaviour and treatment strategy [73]. Radiotherapy, particularly external beam radiation therapy (EBRT) is well established in the treatment of localised PCa [77,78], but is also used in some patients with metastatic burden. Combination treatment options are used in higher risk cases. For example, prostatectomy, followed by radiotherapy is used in high-risk cases. Androgens are key drivers of tumour growth, especially in the early stages of the disease. Consequently, androgen deprivation therapy (ADT) approaches including hormonal therapies to deprive the tumour of a crucial growth factor play an essential role in controlling progression in localised and metastatic disease. When the disease is localised, ADT is commonly used in combination with EBRT [74,75]. ADT including luteinizing hormone-releasing hormone (LHRH) agonists, (leuprorelin, goserelin and triptorelin), have been used to stop androgen production, actuating medical castration [79]. However, they can give rise to tumour “flare” [80]. Whereas gonadotrophin-releasing hormone (GnRH) antagonists (e.g., degarelix, relugolix) can avoid tumour flare [81]. Androgen receptor pathway inhibitors (ARPIs) (e.g. enzalutamide, abiraterone), represent newer hormone treatments that can inhibit androgen production or block the androgen receptor [82]. They play an important role in the management of castrate resistant and metastatic disease [83,84].

**Fig. 3:**
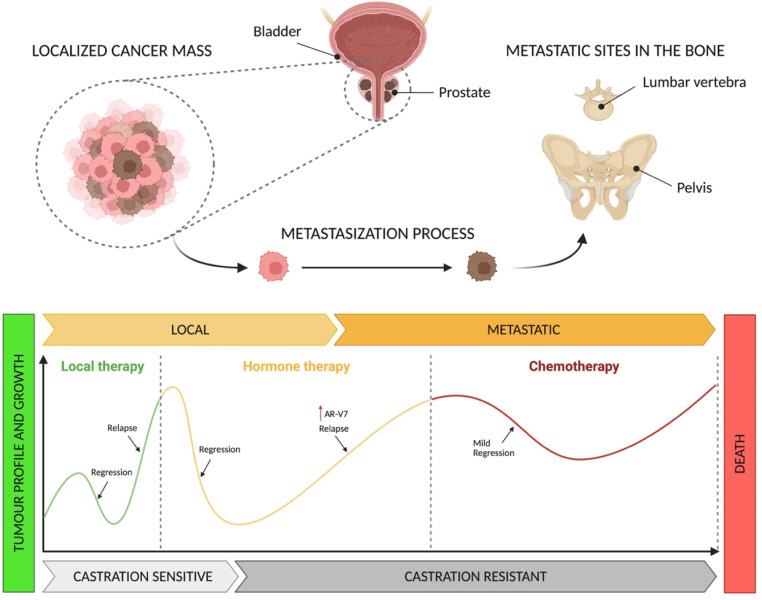
Schematic representation of PCa growth profile and progression with treatment strategies available. Note: this figure is for illustrative purposes only, it does not represent the exact magnitude of change. Created with BioRender.com.

The metastatic spread signals an important stage in disease progression. PCa cells acquire a more aggressive phenotype [85]. Taxane chemotherapy (e.g. docetaxel, cabazitaxel) is part of the standard of care for patients with mPC [86]. Although it is unlikely to cure advanced disease, it can ease symptoms and improve the quality of life for patients (Fig. 3). Combination therapy approaches have been used to treat metastatic hormone sensitive PCa (mHSPC) and metastatic castrate resistant PCa (mCRPC) patients. Primary ADT in combination with ARPI or chemotherapy (e.g. docetaxel) is part of the standard of care in the treatment of mHSPC [87], while a triplet regimen combining ARPI, ADT and docetaxel has been used in patients with high volume disease [78,88].

The alpha particle emitting, radiopharmaceutical, radium-223 (Ra-223) is approved in patients with mCRPC and symptomatic bone metastases [89]. It uses its calcium mimetic behaviour to target actively growing bone [90]. Treatment with radium-223 has been shown to prolong survival and improve quality of life [91]. More recently, there is increasing focus on the use of immunomodulatory treatments. PROVENGE® (Sipuleucel-T, (Dendreon Pharmaceuticals LLC, Seal Beach, California, USA)), a personalised immunotherapy treatment for mCRPC [92,93], is an autologous cell product prepared from the patient’s own antigen presenting cells, which are activated *ex vivo* by culturing with the recombinant fusion protein, PAP-GM-CSF. The protein consists of prostatic acid phosphatase (PAP), an antigen expressed on prostate cancer cells and the immune cell activator, granulocyte–macrophage colony-stimulating factor (GM-CSF) [94]. The cells are then reinfused into the patient and mediate a T-cell immune response [95]. Other innovations include, the FDA approved Pluvicto™ (lutetium Lu 177 vipivotide tetraxetan) (Novartis AG, Basel, Switzerland), the first targeted radioligand therapy for the treatment of progressive prostate-specific membrane antigen (PSMA) positive mCRPC in patients who have already undergone treatment with ARPI and taxane chemotherapy [96]. Newer agents such as PARP inhibitors may be considered for mCRPC patients who have confirmed BRCA gene mutations [[97], [98], [99]]. Despite the introduction of new treatments, optimal treatment is not defined, and the prognosis is poor for patients diagnosed with metastatic spread to the bone [100]. These challenges underscore the need to better understand the heterogeneous disease process and to develop treatments that meet patient specific needs.

## 5 The drug development process: challenges driving new approaches to research

Drug development is a lengthy and multi-phase process which begins with basic research in the laboratory and ends with drug products recognized as safe and effective for public use [101,102]. Throughout drug development, a series of mandatory pre-clinical tests and clinical trials are conducted to acquire information about the chosen drug candidate (e.g., activity, specificity, efficacy and toxicity) [103]. These tests involve in vitro (cell cultures) and in vivo (animals) models prior to transitioning to testing in humans [104]. The process is lengthy and expensive, which is compounded by challenges with respect to the attrition of potential candidates as they progress through the development stages. This is attributed, in part, to misleading experimental observations derived using standard and outdated preclinical models [105]. This failure has been associated with the use of poorly predictive models, particularly monolayer 2D cultures, which poorly replicate the intricate and complex three-dimensional (3D), cellular microenvironment. Three-dimensional models have been proposed to represent a more reliable tool than commonly used monolayer cultures to predict in vivo therapeutic outcomes [106]. Furthermore, by developing more humanized and realistic in vitro models, more in-depth studies of the pathophysiology of malignancies such as PC could be investigated in the lab, while they could also offer greater opportunities for the development of personalized medicines [107,108].

The use of animal models, especially mammals (e.g., mice, rats, pigs etc.), for scientific purposes represents a longstanding practice in biological sciences [109]. Animal offer complex and distinct physiological functions all integrated in a single model and have helped advance the understanding of the biology and pathology of many diseases and influenced the development of therapies [110]. However, these studies are controversial due to ethical concerns regarding animal usage combined with challenges of directly translating results obtained from animal models with the human context. Animal models can substantially differ from the human biology in terms of anatomical layout, the expression of receptors and the responses of the immune system [111], which may limit the understanding of underlying disease mechanisms and their utility in drug development [28]. These differences have also helped spur the demand for innovative and alternative models capable of recapitulating the native cell environment and human context [103]. Compared to animal models, 3D models offer the prospect to control the extrinsic stimuli and environmental factors more tightly. Further, 3D models support the 3Rs principles of replacement, reduction, and refinement [19], sparing the use of animals in research and enabling more ethical and sustainable approaches to be utilised in the drug development. The use of 3D models and alternatives to animal models has been supported by the European Union through a resolution encouraging the transition to the use of new approach methodologies (NAM) in research, regulatory testing and education [112]. However, their translational capability continues to be questioned [12]. Technical and regulatory criteria need to be established, and agreement is needed on how these models will be used in key decision-making processes. Further the conceptual bias needs to be challenged that experimentation with 3D models cannot be fully accepted if the experiments are not conducted in conjunction with animal models [113].

Pivotal in the development of innovative and useful models includes the reproduction of the cells' natural microenvironment to mimic the relevant mechanical and biochemical cues inherent in the healthy and diseased state [114,115]. Successful recapitulation of the natural microenvironment must take account of key factors such as the morphology, the material composition, and the residing cells [116,117]. The importance of these factors and the lack of clear design guidelines have led to the introduction of different 3D models based on different design paradigms, materials systems and manufacturing approaches to achieve the goal.

## 6 In vitro 3D models of bone tissue

To efficiently model mPC in bone, 3D scaffolds need to emulate the hierarchical structure, composition, and the complex, dynamic cell interactions in the bone microenvironment. 3D in vitro models for cell cultivation include those with a 3D artificial support (scaffold-based) and scaffold-free [118]. Typical scaffold-free models include spheroids and organoid self-assemblies, which mimic cellular integration and tissue function to varying degrees [119]. However, they can fail to accurately replicate porous architecture and hierarchical tissue structure inherent in native bone tissue. On the other hand, scaffold-based models constitute an artificial temporary niche for cells and provide structural protection and mechanical strength depending on the specifics of the targeted tissue. Research to date focused on designing 3D archetypes of the bone for modelling mPC, have taken inspiration from the field of bone tissue engineering (BTE) and regeneration. Consequently, scaffold-based models account for the primary 3D modelling approach employed to date.

Like the design of scaffolds for BTE, the target profile of constructs intended for 3D modelling applications should take account of the physical and biological features of the target tissue. These together with considerations related to scalability (if required), reproducible production, and material choice e.g., to ensure biocompatibility and requisite chemical and mechanical properties [120,121] will impact methods used to manufacture scaffolds.

### 6.1 Structural and spatial characteristics of 3D scaffolds

The pore dimensions, porosity and intrinsic geometry of the scaffolds represent important features which determine the mechanical resistance of the scaffolds and impact several cellular processes including the migration of the cells to the scaffold, cell adhesion, colonization and proliferation [122,123]. There is still debate around the ideal pore dimensions of a scaffold intended to replicate the bone, and different pore ranges have been proposed: 100–135 µm [124] and 20–1500 µm [125]. Pore sizes can be categorized as nano-sized (<100 nm), micron-sized (0.1 µm-100 µm) and macro- pores (>100 µm-mm). In general, smaller pores aid in cell entrapment, infiltration and subsequent deposition of new bone while larger pores support fluid, nutrient and waste exchange [126,127].

The dimensions of the pores and their level of interconnection are interdependent with the porosity of the scaffolds. Again, no fixed and optimal porosity value has been defined. However, the porosity of the scaffolds should consider the multilevel porosities inherent in the bone cavities (marrow, lacuno-canalicular and ‘nano-porosity’ at the level of collagen and HA crystals) [128]. Therefore, the porosity, depends on the type of bone considered. Cortical bone is less porous (∼5–20 %) [129,130] compared to trabecular bone, which is highly porous (∼50–90 %) [131]. It has been observed that higher porosity values (70–80 %) [132] can boost the osteogenic differentiation due to enhanced nutrient exchange and oxygenation of the cells [133].

Of the scaffold models developed to replicate the bone, they differ in their dimensions, the materials used and manufacturing techniques. Consequently, it is challenging to directly compare the physical and mechanical properties of different models. Cruz-Neves et. al (2017), used µCT to show that the surface area of cylindrical (diameter: 5.33±0.19 mm; height: 2.34±0.29 mm), polyurethane (PU)-based hydroxyapatite (HA)/collagen scaffolds was 193±2 mm^2^ [134]. Further, the mean microporosity was > 62 %, falling within the range (45 % to 90 %) suitable for successful bone ingrowth [135].

Ambre et al. produced exceptionally porous (> 85 %) HA-nanoclay polymer composite scaffolds, with pores ranging from 100–300 µm for nutrient exchange and micropores (< 10 µm) lining the main pores [136]. Addition of the hybrid HA-nanoclay (10 %) increased the mechanical strength by 94.16 % compared to control polycaprolactone (PCL) scaffolds (1.285±0.212 MPa) and caused a reduction in porosity (from 88.83 % to 86.06 %). Similar observations were observed with poly(lactic-co-glycolic acid (PLGA) polymer scaffolds containing HA. The average porosity of PLGA (72.68 ± 3.64 %) scaffolds reduced with the addition of HA (2 mg) (61.71 ± 2.48 %), however porosity increased to 75.39 ± 9.19 % with further addition of HA (4 mg), which was attributed to the enhanced wettability at the higher concentration. However, increasing the HA concentration led to a reduction in the mechanical strength [137].

The ‘intrinsic geometry’ or the shape (e.g., circular, squared, triangular) of the pores in the 3D scaffold has also been shown to influence several cell parameters such as the cell growth because the cells can ‘sense’ the substrate they are growing on through their cytoskeleton [138]. Particularly, osteoblasts growing in curved surfaces displayed a better growth behaviour and bone tissue formation compared to the cells growing in areas without any or reduced curvature [139]. A recent study indicated that innate ‘sensing’ of stronger curvatures such as hyperbolic or ‘saddle’ surfaces, whereby the total Gaussian curvature (K) K<0, was proposed to translate into faster deposition of bone tissue by osteoblasts [140]. While Ambre et al., 2014 concluded that the presence of lamellar features in HA-clay PCL scaffolds may have signified changes in pore properties and interconnectivity and potentially contributed to enhanced scaffold osteoinductivity [136].

### 6.2 Materials used to produce 3D scaffold models

Scaffolds have been produced using a wide range of materials which can be categorised based on origin (natural and synthetic), or material behaviour (degradable or non-degradable) [121,141,142]. Selection is based on the desired mechanical properties, scaffold architecture and biological relevance [143]. In general, natural polymers including chitosan, collagen, gelatine, and hyaluronic acid are attractive due to their biocompatibility [144], however, when used on their own, they fail to replicate the mechanical properties of the bone and are prone to deformation [145]. Generally, the human cortical bone has a compressive strength in the range of 100–150 MPa, while the trabecular bone is in the range of 0.1–12 MPa, although 30 MPa in some areas [23,146]. Hence, researchers have commonly utilized them in blends with synthetic polymers to help ensure higher mechanical resistance. For example, Poly(l-lactic acid) (P_L_LA), polyvinyl alcohol (PVA), poly-β -hydroxybutyrate (PHB), PU elastomers, poly(3-hydroxybutyrate-co-3-hydroxyvalerate) (PHBV), poly(D,L-lactic acid) (P_DL_LA), PLGA and PCL are some examples used to make scaffolds for tissue engineering and could be explored for 3D model applications [[147], [148], [149]]. Concerning synthetic biodegradable polymers, Li et al., investigated the potential of PCL to enable hydrophobic interactions with self-assembling peptide hydrogels, involving the benzyl group of phenylalanine; thus, enhancing the polymer’s hydrophilicity and promoting stem cell growth and osteogenic differentiation [150]. Compared to PCL, poly(lactic acid) (PLA) exhibits a relatively higher intrinsic hydrophilicity, while PLGA, contains glycolic acid, further enhancing the hydrophilic properties. The ratio of lactic and glycolic acid can be synthetically modulated to fine-tune its final degradation rate. PLGA is widely recognised as a promising biomaterial for BTE, due to its biocompatibility and ability to support osteogenic differentiation [151].

Regarding non-degradable, synthetic polymers, polytetrafluoroethylene (PTFE) is hydrophobic, chemically inert, and characterised by a low, intrinsic porosity, which can be modified using sintering or etching techniques to enhance its permeability and favour cell adhesion [152]. Polymethyl methacrylate (PMMA) exhibits a smooth and hydrophobic surface and has been used in bone cement applications [153]. PMMA has been copolymerised or coated with bioactive materials to enhance osseointegration [153]. Another polymer, polyether ether ketone (PEEK) has shown excellent mechanical properties and chemical stability in bone applications [154]. PEEK has also been modified using laser ablation or plasma etching to introduce microporosity [155]. PU is an elastic and hydrophilic, non-degradable polymer, which is used to support cellular interactions and mechanical resilience. Due to its high tunability and interconnected porous networks it is used in vascular grafts and BTE applications [156].

Additionally, blends consisting of synthetic and/or natural biopolymers with bio-active calcium phosphate ceramics, can be engineered to enhance biocompatibility and optimize scaffold properties (physical, mechanical and biological). In the context of this review, many of the scaffolds produced to model mPC have involved mixing of degradable synthetic polymers and HA, or other biorelevant materials prior to scaffold production. These composites may include micro- or nanosized hydroxyapatite (nHA), β-tricalcium phosphate (β-TCP), carbonated calcium-deficient hydroxyapatite, bioactive glasses, or functional agents (e.g., drugs and biomolecules). The inclusion of calcium phosphate (CaP) containing agents e.g., HA, which are chemically similar to bone mineral, can help promote bioactivity and osteoconduction, while their hydrophilic nature enhances protein adsorption and cell adhesion [149,157,158]. Moreover, these ceramics are highly tuneable, with macropores (100–500 μm) for vascularization. In addition to replicating the compositional environment of bone, the addition of nHA can alter the topology of the scaffold surface favouring cell adhesion onto rougher surfaces consequently promoting the cell colonisation [159,160]. For example, lyophilised collagen-based scaffolds supplemented with HA or glycosaminoglycans were utilized as physiologically relevant testbeds to model bone metastases derived from PC and evaluate the delivery and efficacy of cyclodextrin-based gene therapeutics designed to target metastatic PC cells in the bone [106]. The model was successful in demonstrating that gene silencing was mediated in cancer cells. However, the mechanical strength of the scaffolds peaked at ∼ 6 kPa only when HA was added to the collagenous mix, which, is substantially less than the strength required to mimic the bone (100 kPa minimum, trabecular bone) [129].

### 6.3 Surface modification of 3D scaffolds

The scaffold’s surface can be modified to increase the physico-chemical and structural relevance with respect to the tissue target, while reducing the chances of bodily rejection [161]. Indeed, the scaffold’s biocompatibility and the ability to create optimal conditions for cell colonisation and cell growth can be enhanced by modifying the materials used (pre-production) or the scaffolds (post-production) [162]. Such strategies have been employed in BTE, which represents the closest field of reference for modelling mPC spread to the bone, and in the development of orthopaedic implants [163].

Surface modification has been used to enhance the properties of a range of materials ranging from synthetic, biodegradable materials, to plastics or resins [164]. As mentioned earlier, sintering, laser ablation and plasma etching have been used to tailor the porosity of polymers [152,154]. A common approach includes coating with bioactive materials including surface functionalisation with HA to improve osteogenic potential [153,155]. Coating of the scaffold using one of a variety of physical techniques, e.g., dipping, spin coating, drop coating, layer-by-layer deposition, electrospray and electrophoretic deposition, has been commonly employed to modify the surface properties [165]. Alternatively, the surface can be chemically functionalised, which can produce stronger bonds with biomolecules and is preferred over physical adsorption techniques [166]. In the context of modelling mPC spread to bone, several studies have used PCL scaffolds coated with CaP [167,168]. One study cultured PCL scaffolds under osteogenic conditions to induce matrix mineralisation [167]. Additionally, CaP coating has been achieved using a multi-step process that involved surface activation with sodium hydroxide (NaOH), CaP deposition in simulated body fluid, followed by a post-treatment step in NaOH to homogenise the CaP phase [168].

Surface modification with plasma treatment has been proposed to control the topological or interfacial scaffold properties in a more precise manner [166,169]. For example, Park and colleagues compared the proliferation and differentiation of murine pre-osteoblasts on high density polyethylene (HDPE) scaffolds containing nHA particles against the cell behaviour on HDPE/nHA scaffolds plasma treated with nitrogen [170]. The group found the wettability and roughness of the HDPE/nHA scaffold surfaces were enhanced because of the etching effect caused by plasma treatment. The combination of nHA and surface treatment enhanced the initial attachment, proliferation, and differentiation of murine pre-osteoblasts and scaffolds with plasma treatment showed the highest bioactivity compared to controls.

## 7 3D models and fabrication techniques

Different fabrication techniques have been employed to produce scaffolds, and these can be broadly categorised into i) conventional fabrication methods and ii) additive manufacture (AM) including 3D printing (3DP) techniques.

### 7.1 Conventional fabrication methods

**Fig. 4:**
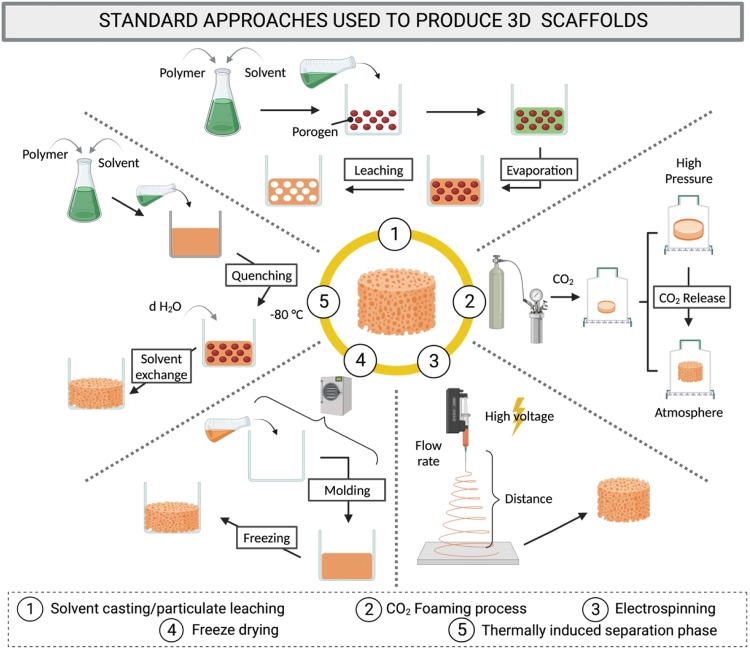
Illustration of the conventional methodologies used to produce scaffolds. (1) Solvent casting/particulate leaching; (2) high pressure CO_2_ foaming; (3) electrospinning; (4) freeze drying (lyophilisation) and (5) thermally induced phase separation. Created with BioRender.com.

A series of conventional methods have been employed to produce scaffolds [171]. Examples include gas foaming, particulate leaching, electrospraying and electrospinning are depicted in Fig. 4. Table 1 provides a brief description of some of these conventional techniques.

**Table 1:** Overview of conventional fabrication methods that have been employed to produce 3D scaffolds.

Technique	Method description	Advantages	Disadvantages	Ref
Solvent casting/particle leaching	A solution of polymer in organic solvent is casted or poured in a mould containing a porogen e.g., salt consisting of particles of a specific diameter. When the organic solvent evaporates, the polymer matrix is left in the mould and incorporates the salt porogen, which is subsequently eliminated by leaching in aqueous media.	• Simple and straightforward • Low cost • Scalable • Controlled porosity only through selection of particle dimension and shape • High porosity	• Presence of residuals of cytotoxic organic solvents • Limited scaffold thickness • No use of water-soluble materials	[172,173]
Gas foaming	Two separate phases characterise the high pressure/supercritical gas foaming process, which is performed using a blowing agent, e.g., CO_2_. i) Under elevated pressure CO_2_ dissolves in the polymer while ii) the pressure drop during depressurization or at high temperatures causes bubble nucleation, generating pores in the polymeric matrix.	• No use of organic solvents • No use of high temperature • Allow use of bioactive molecules	• Poor control over pore interconnectivity • Limited range of pore size • Costly • Low mechanical properties	[[174], [175], [176]]
Lyophilisation/freeze drying	The sample is first prepared by either dissolution or suspension of the polymer in water or organic solvents followed by emulsification with water. The slurry is then frozen and water in the form of ice crystals is removed by sublimation, by reducing the pressure with application of vacuum and changes in temperature.	• Does not require extra processing steps • Scalable	• Use of organic solvents may limit the addition of bioactive molecules • Difficult control over pore distribution if cycle is not controlled • Lengthy process • Low mechanical properties • Energy consumption	[171,177,178]
Electrospinning	Spinning consists of the rapid ejection of fibres though a specific gauge needle, at a certain flow rate and distance to target, by continuous application of high voltage on a solution of polymer alone or polymer mixed with added materials.	• Control over diameter and fibre morphology • Straightforward • Low cost	• Poor cell infiltration • Low mechanical strength • Possible presence of residual solvent	[[179], [180], [181], [182]]
Thermally induced phase separation	A homogenous polymer solution is de-mixed into a multi-phase system either solid-liquid (polymer/solvent mixture) or liquid-liquid (polymer/solvent/non-solvent mixture) through the change in temperature. A wide variety of scaffolds with different pores and porosities can be produced.	• High porosity • High interconnectivity	• Poor control over internal structures and uniformity • Low reproducibility • Limited materials	[[183], [184], [185]]

To date, of the scaffolds available for modelling cancer and PC in the bone (Table 2), the majority has been produced using conventional techniques.

**Table 2:** Summary of the different 3D bone-like models available to model mPC spread to the bone.

Material(s)	Technique	Cell line(s)	Objective	Ref
Collagen-HA & GAG	Lyophilisation	PC-3 or LNCaP	• Develop physiologically relevant bone metastatic niches • Test nanoparticle-mediated siRNA delivery for gene regulation	[106]
HA clay-PCL (10 % and 20 % w/w)	Lyophilisation	PC-3 or MDA PCa-2b + MSC	• Develop biorelevant models of PCa bone metastases with cells exhibiting different metastatic potential • Display mesenchymal to epithelial transition • Recapitulate blastic and lytic features of bone lesion	[186]
				[187]
Gelatine micro-ribbon scaffolds	Spinning + Lyophilisation	Differentiated human MSCs + osteoclasts + PC-3 or LNCaP	• Establish spatially patterned 3D cocultures to mimic clinical features of bone metastases (epithelial to mesenchymal transition, cancer aggressiveness, bone remodelling) • Integrate 3D models with single-cell RNA sequencing • Model properties of bone lesions	[188]
nHA + PLGA	CO_2_ foaming + porogen leaching	hFOB 1.19 + PC-3	• Model metastatic PCa niche in bone • Use model for drug testing • Evaluate cell behaviour	[137]
PU sponges (Reticel, Belgium) + nHA & collagen	Coating + chemical-based crosslinking	MC3T3-E1 + PC-3 or LNCaP	• Establish a bone model to understand mechanisms of PCa metastases • Understand the role of SPARC protein in the oncogenesis of PC	[134]
mPCL-TCP (Osteopore, Singapore)	FDM	PC-3 or LNCaP + patient-derived osteoblasts	• Develop a tissue-engineered bone model to assess the interplay between PCa and osteoblasts cells	[189]
mPCL - CaP	Melt electrowriting	PC-3 + hMPCs	• Develop a tissue-engineered bone construct • Recapitulate the characteristics of bone including a functional marrow niche • Investigate the interaction and homing mechanisms between PCa and MSCs	[168]
		PC-3 + CAF + hOBs + HUVEC + BVEC	• Study the mechanisms of PCa metastases • Reproduce a spontaneous metastasis inclusive of cell infiltrates	[167]
		LNCaP, C4–2B or PC-3 + hOBs/osteocytes	• Engineer a 3D bone model replicating an osteoblastic bone lesion • Evaluate cellular response to androgen-deprivation therapy	[190]

Blood vessel endothelial cells (BVEC); calcium phosphate (CaP); cancer associated fibroblasts (CAFs); glycosaminoglycans (GAG); human mesenchymal progenitor cells (hMPCs); human osteoblasts (hOBs); human umbilical vein endothelial cells (HUVEC); hydroxyapatite (HA); mesenchymal stem cells (MSCs); medical grade polycaprolactone (mPCL); poly(lactic-co-glycolic) acid (PLGA); polyurethane (PU); tricalcium phosphate (TCP)

**Fig. 5:**
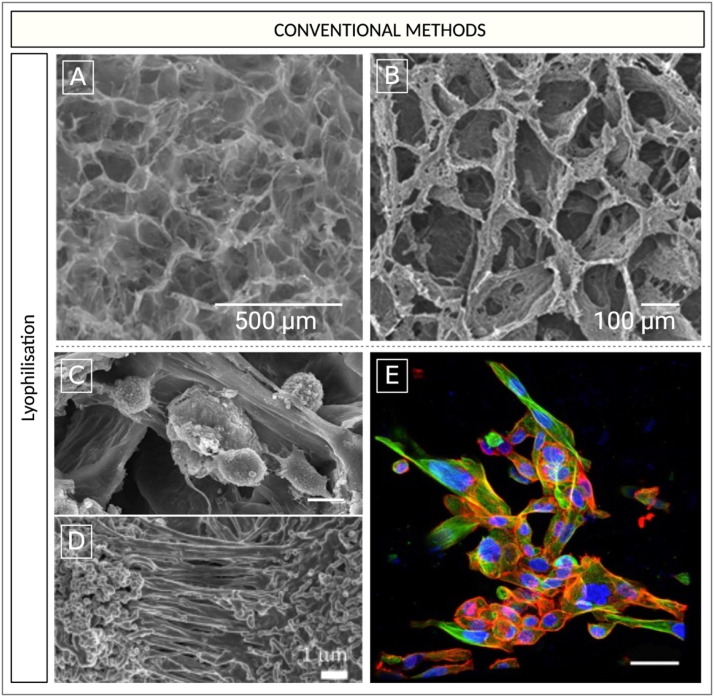
SEM images of scaffolds produced with conventional methodologies. A) Collagen-HA and glycosaminoglycans (GAG) scaffolds produced by Cunniffe et al., 2010 using lyophilisation and employed by Fitzgerald et al., 2015. Scale bar 500 μm. B) 10 % HA clay-PCL scaffolds produced using lyophilisation by Ambre et al., 2015 and tested by Molla et al., 2019 and 2020 [186,187]. Scale bar 100 μm. SEM micrographs displaying: C) the sequential culture of PC-3 and MSCs presenting with disorganized cellular aggregates exhibiting rough surfaces (Scale bar 10 μm), D) PC-3 and MSCs rich in ECM (Scale bar 1 μm). E) Confocal microscopic images of immunostained α‐tubulin (green), F‐actin (red), and nuclei (blue) in sequential culture of PC-3 and MSCs. Scale bar 50 μm. Images adapted with permission from (A) Cunniffe et al., 2010 [194] (B), Ambre et al., 2015 [136] and (C-E) Molla et al., 2020 [187]. Figure created with BioRender.com.

In particular, lyophilisation, as a manufacturing technique, has been employed in several studies [106,191,192] (Fig. 5 A, B), perhaps owing to the potential for scalability and the production of many scaffold units simultaneously [193]. Molla and colleagues lyophilised a slurry of HA-PCL to obtain bone bio-mimetic scaffolds that were utilised in several studies to reproduce the metastasising behaviour of PC cells including MDA PCa-2b (low metastatic) and PC-3 (highly metastatic) cultured with MSC, in vitro [186,187] (Fig. 5 B). Experimentally different phases of PC metastasis were observed. Notably, the authors observed that the more aggressive PC-3 cell line produced an osteoblastic response, which contrasts with the effects of PC-3 cells observed in other studies [187]. While excessive osteolysis was observed in the presence of the less metastatic MDA PCa-2b. Furthermore, both PC-3 and MDA PCa-2b, have previously been shown to demonstrate the mesenchymal-to-epithelial transition, a process by which cancer cells increase their colonisation capability at distant sites [186].

The gas foaming/porogen leaching technique, was used to produce nHA-PLGA mixed 3D scaffolds as bone biorelevant (trabecular bone) niches. Modelling of metastatic insult was produced by co-culturing highly invasive mPC cells, PC-3 with osteoblast cells hFOB 1.19. The study concluded that the use of the PLGA copolymer blended with nHA, together with the manufacturing method altered the scaffold’s microenvironmental cues. Consequently, the malignant profile of the PC-3 cells was enhanced at the expense of bone cells contributing to the development of clinically relevant niches of mPC in the bone [137].

A major limitation associated with lyophilisation and other conventional fabrication techniques is the limited control over the pore structure, geometry, and interconnectivity in 3D scaffolds. Conventional techniques including lyophilisation can produce heterogenous structures [171] and can give rise to scaffolds with intra- and inter-batch variability [195]. Poor control over the scaffold’s porous structure and morphology during the manufacturing process can have ramifications for several cellular mechanisms including cell adhesion, colonization, proliferation and matrix deposition [196,197].

### 7.2 Additive manufacturing (AM) techniques

**Fig. 6:**
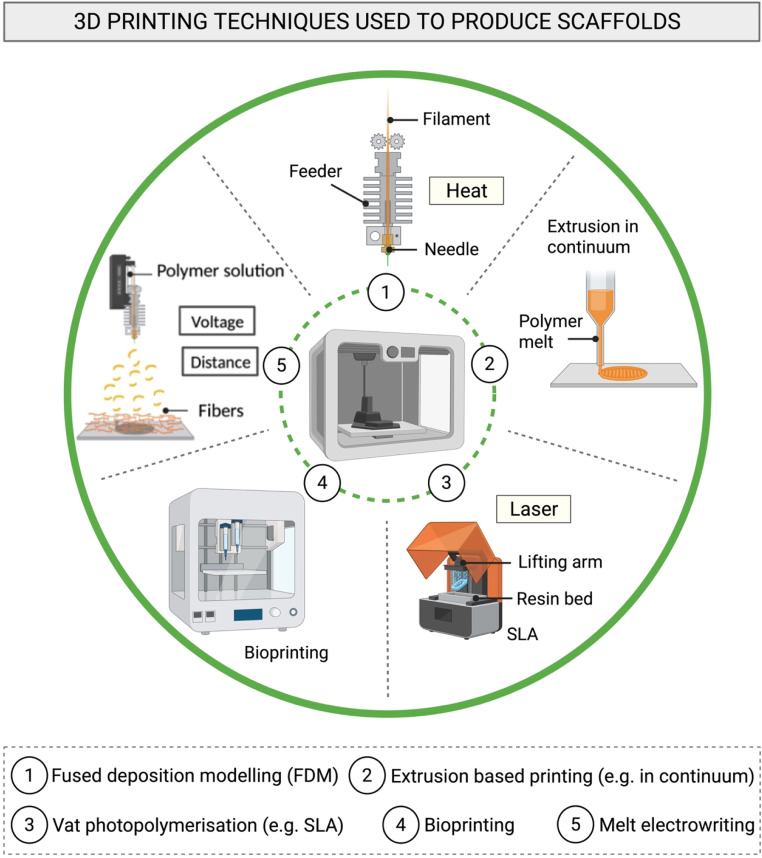
Illustration of the AM techniques used to produce scaffolds. (1) Fused deposition modelling (FDM); (2) extrusion-based 3D printing techniques: extrusion in continuum; (3) Vat photopolymerisation (e.g. SLA); (4) bioprinting and (5) melt electrowriting. Created with BioRender.com.

Additive manufacturing (AM) techniques or 3DP have been employed as an alternative fabrication approach to address the problem of scaffold variability and to produce more sophisticated constructs. AM represents the concretization into a solid manufact of an abstract, virtual design concept established a priori using computer-aided design (CAD) software such as AutoCAD® [198]. Constructs can be 3D printed depending on the working principle of the device e.g., deposited in layer-by-layer modality [199,200] or volumetric photopolymerisation [201]. AM encompasses a series of techniques capable of producing high quality constructs, although the precision and resolution can vary depending on printer type. Extrusion-based printing techniques, melt electrowriting, fused deposition modelling (FDM), stereolithography (SLA) and 2-photon polymerization (2PP) (a more advanced form of SLA), represent some of the 3DP methods investigated, Fig. 6 [171].

Table 3 summarizes the main AM techniques and gives a brief description of the methods involved. There has been little in the way of research utilizing AM approaches to model mPC in the bone [[202], [203], [204]], with only a few studies published to date. In the context of BTE and PCa modelling in 3D, FDM has been the most utilised technique followed by SLA, while there has been less research published using 2PP.

**Table 3:** Comparison of different additive manufacturing techniques in scaffold fabrication.

Technique	Advantages	Disadvantages	Resolution range	Ref
Extrusion based printing (i.e. in continuum, inkjet etc.)	• Enables fabrication of complex 3D shapes with controlled biomaterial placement. • High resolution range, supporting the creation of systems with complex geometries.	• Extensive rheological assessment required to ensure 3D shape fidelity. • Biocompatibility issues due to curing agents and/or crosslinking mechanisms.	5–200 µm	[205,206]
Melt electrowriting	• High printing resolution and control, allowing precise replication of bone tissue features. • Mechanical property tuning, enabling the simulation of bone mechanical anisotropy.	• High complexity and equipment costs. • Limited to fibrous scaffold fabrication, requiring additional processing for bulk 3D shapes.	2–5 µm (fibre dimension)	[207,208]
Fused Deposition Modelling	• Compatible with a wide range of thermoplastic polymers. • Straightforward setup and relatively fast operation.	• Lower printing resolution and poorer surface characteristics compared to other 3DP techniques. • Weak interlayer bonding, caused by inconsistent material deposition, leads to mechanical anisotropy.	50–200 µm	[209,210]
Stereolithography	• High resolution and printing accuracy, facilitating the fabrication of complex geometries and surface features. • Favourable for thermosensitive materials, due to minimal applied thermal stress.	• Higher equipment operational costs. • Limited material options, predominantly photopolymer resins. • Post-processing is necessary to enhance mechanical properties.	up to 10 µm	[206,211]
2 – photon polymerisation	• High spatial resolution and fine features, closely mimicking the ECM. • Compatibility with various photopolymerisable materials.	• Complex technique with low scalability (to date). •Restricted to photopolymerisable materials.	0.2 µm	[212,213]

**Fig. 7:**
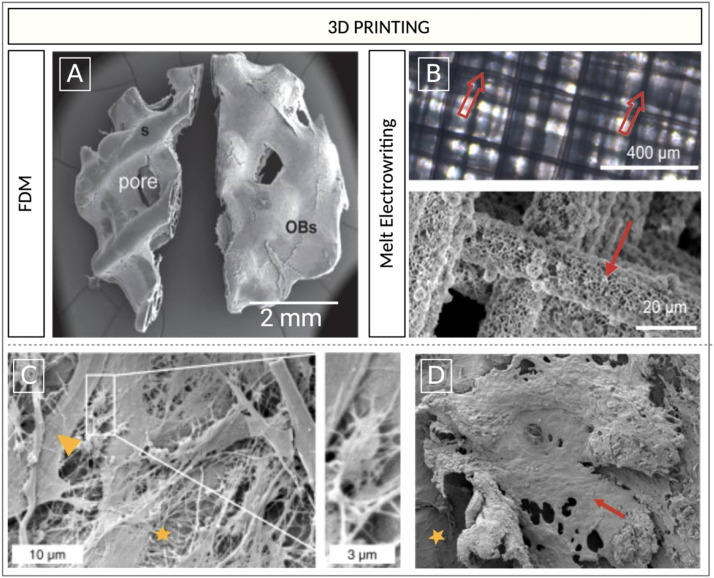
A) SEM images of FDM printed mPCL -TCP scaffold (Osteopore®, Singapore) wrapped in osteoblast sheets [189], scale bar 2 mm. B) Light micrograph of melt electrowritten PCL-based scaffold produced by Bock and colleagues (open arrows point to fibres) and SEM micrograph of CaP treatment (solid arrow) [190], scale bars 400 and 20 μm. SEM imaging showing: C) ECM deposition (asterisk), osteoblastic cells (arrow head), and osteocytic cells (inset) on bioengineered human osteoblast-derived microtissue (hOBMT) (asterisk), (scale bars 10 and 3 μm) and D) PCa cells (LNCaP) attached and aligned onto the surface of the hOBMT (red arrow), adhering to each other and to the hOBMT (asterisk) to form bulk micro-metastatic aggregates by 21 days of cell culture. Images adapted with permission from Sieh et al., 2010 [189] and Bock et al., 2019 [190]. Figure created with BioRender.com.

The Osteopore®, (Singapore) scaffold is printed using FDM (Fig. 7 A) and made of medicated polycaprolactone tricalcium phosphate (mPCL-TCP) [189]. The constructs were wrapped in mineralized osteoblast sheets to generate bony constructs and have been used to cultivate different PCa cells (PC-3, LNCaP). An increase in the production of MMP-9 in scaffolds seeded with PC-3 cells was observed consistent with previous research observations that highlight their aggressive metastatic behaviour [189].

Melt electrowriting has been employed in several studies to model mPC in the bone [167,168,190]. In one study, a PCL based microfiber scaffold was coated with calcium apatite (Fig. 7 B-D) and used as a niche for human osteoblasts. The archetype served as a testbed to evaluate the effect of ADT on both osteoblasts and different types of PC cells. It succeeded in replicating the clinical characteristics of a pure osteoblastic bone lesion induced by PC and the authors claimed that it provided a valid substrate to study PC pathophysiology [190].

Most of the scaffolds produced to replicate the bone environment using AM are the product of straightforward designs and do not closely approximate the bone architecture. Ahangar and colleagues [214] printed different PU-PVA, nano-porous, scaffolds (0.6 mm in height and 5 mm in diameter) via FDM for the localized delivery of doxorubicin in bone defects arising from surgical resection of mPC in the spine [214]. The scaffolds were manufactured via a hybrid method. AM was first used to obtain a monolith scaffold, while PVA was later washed out afterwards with water to create a spongy structure [214]. Other designs consist of layers of perpendicular or wavy struts serially deposited to create a network [215,216] or represent the orthogonal repetition of the same polygonal face (e.g., triangle, square, hexagon etc.) [217]. Attempts to mimic the bone appear to have concentrated on the choice of materials rather than recapitulating bone physical architecture in the 3D constructs [218]. Therefore, there is a need for alternative and structurally relevant CAD designs to obtain AM scaffolds for PC modelling.

### 7.3 3D bioprinting in bone cancer tissue models

#### 7.3.1 Introduction

Among the various 3DP techniques, bioprinting, which allows for printing of cell-based inks (e.g., bio-inks), has garnered increasing attention to reconstruct cancer niches [219], including those of breast [220], liver [221], ovarian [222] and prostate [223]. 3D bioprinting seeks to enhance the spatial, mechanical and biochemical properties of 3D models by developing consistent 3D structures, using various biomaterials and cells [224], thereby recapitulating tissue environments [225] and enabling the in vitro investigation of biologically triggered phenomena with unprecedented control and relevance. This approach allows researchers to explore processes such as new vascularisation, cellular crosstalk, cellular proliferation and migration, phenotype development, cancer progression and tumorigenesis, as well as drug response in different stages of cancer development [226,227]. Hence, maintaining uniformity in geometrical features and bioink consistency are critical to developing physiologically relevant cancer-like 3D models and ensuring thorough investigations.

Research on mPC, is still at an early stage, likely due to the challenges involved in replicating the tumour microenvironment and the interactions associated with bone-metastasis. The low viscosity of the printing bioink required to preserve cell viability, contrasts with the high mechanical requirements of bone tissue, which complicates the bioprinting process [228]. Therefore, bioprinting models that simulate mPC bone tissue remain an emerging research focus requiring additional investigation to balance the need for biologically relevant models with the challenges of manufacturing.

#### 7.3.2 3D bioprinting techniques applied for bone cancer research

**Fig. 8:**
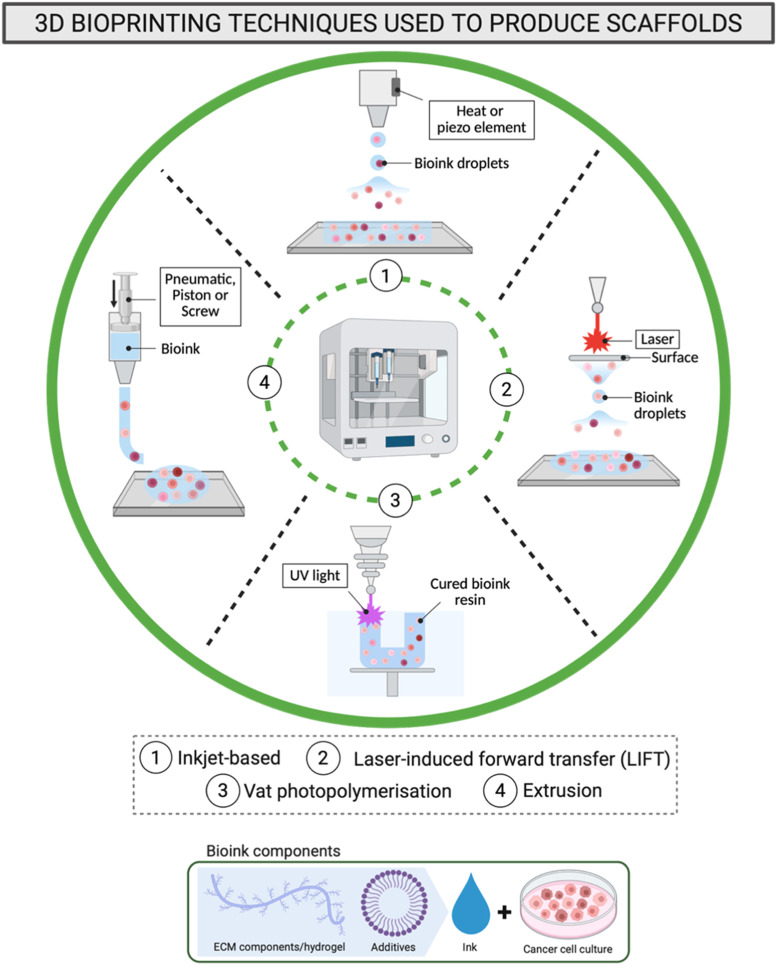
Illustration of 3D bioprinting techniques used to produce scaffolds. (**1**) Inkjet-based bioprinting; (**2**) laser-induced forward transfer (LIFT); (**3**) vat photopolymerisation (VPP) and (**4**) extrusion-based. Created with BioRender.com.

Several 3D bioprinting techniques have evolved for the fabrication of artificial tissues. Inkjet-based, laser-induced, vat photopolymerisation (VPP) and extrusion-based (Fig. 8) are the most recent bioprinting techniques being applied in cancer research. Each bioprinting technology presents distinct strengths and limitations, depending on its specific application and the ability to accurately replicate the target tissue microenvironment. The consistency of printed bioinks is critical in determining the appropriate bioprinting technique [225,227].

Inkjet bioprinting, using “drop-on-demand” methods, enable the precise and controlled deposition of bioink into tiny droplets ranging from 1–100 μL onto a substrate or scaffold following a predetermined pattern [229]. Droplet formation is actuated either by a thermal mechanism, which creates and discharges an internal vapor bubble [230], or by a piezoelectric element that creates mechanical pulses to expel the droplet. Therefore, low viscosity bioinks (<1×10^6^ cells/mL, 3.5–12 mPa·s) may be essential to assure a reliable printing process [230]. This high-resolution technique is efficient, producing minimal bioink waste and is easily scalable [231]. Nevertheless, the mechanisms involved in droplet formation may potentially disrupt cell membranes, thereby releasing fragments, which accumulate inside the nozzle, leading to clogging and 3D models with compromised mechanical integrity.

Laser-induced forward transfer (LIFT) bioprinting represents a dynamic process of material transfer between the donor and the receiving substrate generated by a focused laser pulse (193 to 1064 nm) [232]. This technique offers a highly controlled and precise layer-by-layer deposition of biological substances, permitting cell densities up to 10^8^ cells/mL [233] in a high-fidelity and complex architecture, achieving a 40–100 μm printing resolution. Although LIFT can decrease shear stress, cell survival may still be affected due to the high-energy laser pulses and rapid mechanical forces involved. Additionally, the high complexity and cost of the equipment can limit the accessibility and scalability of the process [234].

VPP based bioprinting utilises photosensitive bioinks, which are typically hydrogel-based formulations containing living cells and biological materials constituents. These bioinks solidify or crosslink when exposed to UV–VIS wavelengths. Materials exhibiting low viscosity (range of 0.25 to 10 Pa·s) and cell density are considered suitable, to minimise light scattering [235] and facilitate removal of the uncrosslinked bio-ink [236]. This technique allows the rapid fabrication of highly precise and intricate structures, offering superior resolution up to 25 μm by curing entire layers simultaneously, ensuring a high cell survival (up to 95 %) [237]. Micro-structures such as tortuous formations and hollow cavities can be created resembling mosaic architectures [238]. Despite its high spatial fidelity and rapid fabrication capability, VPP bioprinting can expose encapsulated cells to photothermal stress, as UV light and heat may compromise their viability, thereby hindering its large-scale adoption in clinical tissue fabrication [239,240]. Recent developments in volumetric bioprinting (VBP), however, have mitigated some of these limitations through a layerless, tomographic projection process that enables ultrafast and simultaneous resin photopolymerisation. This continuous bioprinting mode facilitates the formation of complex, anisotropic, and support-free structures with superior fibrous internal characteristics, while maintaining compatibility with highly viscous bioinks (>10 Pa·s), thereby improving cell inclusion uniformity, advancing the design of mechanically robust, next-generation tissue scaffolds [241].

The extrusion-based process typically involves the continuous material flow through a syringe system and its efficient attachment on a print bed, while maintaining its consistency and shape during the printing path. This technique is based on the application of force over a surface which is controlled by a screw-driven (rotational force), a piston-driven (vertical force), or a pneumatic regulated (air power) process [242]. Moreover, it affords micro-resolution, approximately 100–500 μm, is effective in dispensing high viscosity biomaterials (≈ 10^4^ Pa·s) [243], and cell concentrations exceeding 1×10^6^ cells/mL [205]. However, the force applied during extrusion should be informed by the rheological properties of the bioink, particularly since the shear stress exerted on the cells during material deposition can subsequently affect cell viability in the final product [244].

The fabrication of 3D, complex geometries to replicate tissue-like structures has encountered challenges related to shape integrity and long-term mechanical stability, prompting the exploration of embedding technologies. The FRESH (Fused Reversible Embedding of Suspended Hydrogels) bioprinting technique facilitates the extrusion of a bioink into a thermoreversible support medium [245]. This support material solidifies to sustain and prevent the 3D structure from potential collapse or deformation and can subsequently be liquified to release the final printed construct. Moreover, this method contributes to the accurate deposition of cells and biomaterials while protecting cells from shear stress, thereby potentially improving overall cell viability [246]. However, the technique involves complicated pre- and post-processing steps, including optimisation of the support material composition and its subsequent removal, which may pose challenges to the accessibility and scalability of the technique [245,247].

#### 7.3.3 Key bioprinting parameters for developing bone cancer tissues

Critical attributes of bioink composition impact the 3DP process (e.g., printing efficiency) and the final quality characteristics e.g., 3D shape and fidelity, cell survival and bioactivity [248]. In particular, the bioink’s profile is related to the chemical characteristics and molecular weight, the ratio of concentration of both the biomaterials [249] and cells (e.g., 1×10^6^ cells/mL, corresponding to ∼5 % of the final bioink volume, for efficient printing extrudability) [250], and the crosslinking process [251]. Mechanical and rheological properties of bioinks provide important metrics for assessing the shape integrity of the structure and impact nutrient distribution to cells [252]. For instance, high viscosity inks may cause cell membrane disruption, and stiffer structures impede the fast and efficient diffusion of nutrients through the matrix, leading to lower cell survival rate [253].

A variety of materials has been used for the fabrication of 3D printed models, as discussed in Section 6.2. In terms of bioprinting it is important to ensure printability efficiency and cell viability. Collagen and gelatine have emerged as promising candidates for replicating the natural forms and structures of bone tissue [254]. Moreover, alterations in the mineralised, nanocrystalline, HA (∼75 % wt.) phase are observed in cancerous bone tissue, reflecting complex remodelling processes provoked by tumour-induced interactions [255,256]. Therefore, strategies to fabricate mineralised 3D scaffolds have focused on either surface grafting of micro- or nanoparticles or their incorporation into inks to create osteomimetic constructs. Bioactive ceramics have been shown to interfere with the tissue environment by releasing ions (e.g., Na, Ca, Mg, P etc.) and promoting cell-signalling [257]. Furthermore, the surface energy of ceramics and the topographical characteristics of the micro- and nano-particles can enhance cell adhesion and spreading, contributing to their proliferation and efficient deposition into the printed matrix [258]. Additionally, as outlined earlier in Section 6.2 composites of bioceramics and biopolymers can be employed to significantly enhance structural properties, resulting in more cohesive and geometrically precise constructs compared to hydrogel-based alternatives [259]. However, an efficient recreation of an ideal cancerous bone tissue structure has not been published in the literature to date, likely due to the complexity of the native tissue, the intricate nature of the bioprinting process, which involves the complex interplay of multiple physical and biological parameters.

## 8 Outlook

Various bone scaffolds have been fabricated using 3DP to simulate sarcomas [260] and model metastatic breast cancer spread to bone [261,262]. However, 3D bioprinted models recapitulating mPC, remain underexplored. Nevertheless, essential elements for creating in vitro 3D printed cancer models have been investigated; this paves the way to advance mPC models related to bone. From a technological perspective, the resolution of printing techniques is crucial for accurately replicating the in vivo tumour structures, while the incorporation of bioactive ceramics enhances cell tunability in response to internal and external stimuli.

Despite the inherent challenges in replicating the complex variability of phenomena within the "vicious cycle" of cancer metastases, emerging strategies are focusing on optimizing and refining key aspects of bone cancer dynamics. The utilisation of predictive models, such as the application of machine learning and artificial intelligence in 3D bioprinting process (e.g., selection of biomaterials, cell seeding, printing parameters, etc.), holds significant potential in accurately recreating the tumour microenvironment. Further it advances the prospects of personalised evaluation of disease progression and treatment efficacy. The use of more sophisticated designs incorporating biomimetic materials (e.g., mineral and organic components), alongside the ECM complexity of bone tissue, and the incorporation of various cell lines, will improve the constructs biomimicry and lead to the creation of more robust tissue models. Furthermore, the development of an advanced 3D architecture designed to promote angiogenesis and replicate vascular networks characteristic of cancerous tissue will facilitate a deeper understanding of intravasation and extravasation phenomena. In depth studies evaluating oxygen supply, nutrient diffusion, abnormal blood vessel formation, and tumour progression, will provide greater insights into disease pathogenesis at different stages of progression and the efficacy of therapeutic strategies.

## 9 Conclusions

There is a need to enhance the prognostic outlook for patients suffering from mPC spread to the bone. Understanding the interplay between PCa cells and bone cells in biologically relevant in vitro 3D models can give insights into how the cancer arises and progresses. Further biomimetic, 3D models can potentially support the development of much needed effective drug treatments. Additive manufacturing techniques, including bio-printing, offer great potential to generate controlled and detailed 3D models with tailored biological, physical and mechanical properties. However, integrating established knowledge of biological phenomena and inherent properties of native tissues with design optimisation and advanced manufacturing technologies is necessary to faithfully replicate the cancer environment in vitro. Indeed, current trends in machine learning may prove beneficial in advancing scaffold design. The adoption of 3D scaffolds and alternatives such as bio-printed tumour microenvironments, and spheroids are being supported by regulatory agencies globally to address the limitations of 2D cultures and to support animal alternatives and 3Rs initiatives. However, guidance and standardisation in terms of materials and manufacturing protocols are necessary. To help close these gaps, this review has contextualized the metastasization of PCa to bone alongside a critical review of the key parameters, the materials and methodological approaches to guide the design of 3D bone models, to aid researchers in advancing the field.

## CRediT authorship contribution statement

**Annachiara Dozzo:** Writing – review & editing, Writing – original draft, Visualization, Conceptualization. **Aikaterini Dedeloudi:** Writing – review & editing, Writing – original draft. **Dimitrios Lamprou:** Writing – review & editing, Supervision. **Caitriona M. O’Driscoll:** Writing – review & editing, Resources. **Katie B. Ryan:** Writing – review & editing, Writing – original draft, Visualization, Supervision, Resources, Project administration, Methodology, Funding acquisition, Formal analysis, Conceptualization.

## Declaration of competing interest

The authors declare that they have no known competing financial interests or personal relationships that could have appeared to influence the work reported in this paper.

## Data Availability

No data was used for the research described in the article.
